# Antifungal Effect of Hydrogen Peroxide on Catalase-Producing Strains of *Candida* spp.

**DOI:** 10.1155/S1064744995000354

**Published:** 1995

**Authors:** Bryan Larsen, Sandra White

**Affiliations:** 1 Department of Obstetrics and Gynecology Marshall University School of Medicine Huntington WV USA; 2 Department of Microbiology, Immunology, and Molecular Genetics Marshall University School of Medicine 1801 6th Avenue Huntington WV 25703 USA

## Abstract

*Objective:* Clinical isolates of *Candida* were tested for the presence of catalase and susceptibility to hydrogen peroxide.

*Methods:* MIC was tested by broth dilution technique and catalase was determined by a spectrophotometric procedure.

*Results:* All 38 strains tested were inhibited by hydrogen peroxide in concentrations ranging from 4.4 to 88 mM/l, with non-albicans isolates generally requiring higher concentrations of hydrogen peroxide for inhibition. Growth media consisting of glucose and protein diminished the antifungal effectiveness of hydrogen peroxide, as did the presence of hemoglobin, in incubation mixtures. However, hydrogen peroxide exerted greater inhibition at pH 4 than at pH 7. Although all *Candida* isolates tested possessed catalase, there was no apparent correlation between the catalase activity of individual isolates and the minimal antifungal concentration of hydrogen peroxide.

*Conclusions:* This study suggested that, despite the production of catalase by vaginal microorganisms, hydrogen peroxide may exert a regulating influence which may be further modified by the proteins found in the vaginal milieu.


Infectious Diseases in Obstetrics and Gynecology 3:73–78 (1995)
© 1995 Wiley-Liss, Inc.

### Antifungal Effect of Hydrogen Peroxide on Catalase-Producing Strains of *Candida* spp.

**Bryan Larsen and Sandra White**

*Departments of Obstetrics and Gynecology (B.L., S.W.) and Microbiology, Immunology, and Molecular Genetics (B.L.), Marshall University School of Medicine, Huntington, WV*

Abstract

***Objective:*** **Clinical isolates of *Candida* were tested for the presence of catalase and susceptibility to hydrogen peroxide.**

***Methods:*** **MIC was tested by broth dilution technique and catalase was determined by a spectrophotometric procedure.**

***Results:*** **All 38 strains tested were inhibited by hydrogen peroxide in concentrations ranging from 4.4 to 88 mM/l, with non-albicans isolates generally requiring higher concentrations of hydrogen peroxide for inhibition. Growth media consisting of glucose and protein diminished the antifungal effectiveness of hydrogen peroxide, as did the presence of hemoglobin, in incubation mixtures. However, hydrogen peroxide exerted greater inhibition at pH 4 than at pH 7. Although all *Candida* isolates tested possessed catalase, there was no apparent correlation between the catalase activity of individual isolates and the minimal antifungal concentration of hydrogen peroxide.**

***Conclusions:*** **This study suggested that, despite the production of catalase by vaginal microorganisms, hydrogen peroxide may exert a regulating influence which may be further modified by the proteins found in the vaginal milieu.** © 1995 Wiley-Liss, Inc.

Key words

Normal vaginal flora, vaginal bacteria, lactobacilli

For many years, lactobacilli have been considered to be an important part of the vaginal microbial ecosystem, often credited with maintaining vaginal health by inhibiting other microbial species.[1] Clinical evidence indicates an association between the presence of the symptoms of bacterial vaginosis, characterized by an altered vaginal flora with increased numbers of *Gardnerella vaginalis* and various anaerobic species, concomitant with decreased lactobacillus colonization, obvious even on Gram-stained smears.[2] This apparently protective effect was originally attributed to acid production by the lactobacilli, but even in the early part of this century some investigators cast doubt on the role of lactobacilli in producing vaginal acidity.[3,4] In recent years, an alternative explanation has been set forth. Several investigators have indicated that some strains of lactobacilli are able to generate hydrogen peroxide and that its production by these organisms accounts for their beneficial effect.[5–8]

Despite the apparent importance of lactobacilli to the composition of the vaginal flora, the diversity of bacterial species at this site is well known, including, in addition to hydrogen-peroxide-producing lactobacilli, both catalase-producing and non-catalase-producing organisms.[9] It is not clear why organisms lacking catalase persist in the presence of hydrogen peroxide producers or why catalase-negative bacteria persist in the presence of hydrogen-peroxide-producing bacteria. Studies of the vaginal flora have indicated that lactobacilli and *Candida* are frequently coisolated.[10] Because *Candida albicans* contains a catalase,[11] the present study was undertaken to determine its relationship to the

Address correspondence/reprint requests to Dr. Bryan Larsen, 1801 6th Avenue, Huntington, WV 25703.

Received January 23, 1995

Accepted June 5, 1995

Basic Science Article hydrogen peroxide that may be present in the vaginal microenvironment.

### MATERIALS AND METHODS

### Microorganisms

Vaginal cultures were obtained from pregnant patients at their initial prenatal visits. The specimens were plated on BIGGY (BBL, Cockeysville, MD) agar[12] and returned to the laboratory. *Candida* isolates from these vaginal specimens were preliminarily identified by brown colonies on the BIGGY agar. Individual isolates were verified as fungal organisms by microscopic morphology. The ability of each isolate to produce germ tubes was tested in human serum. Germ tubes were produced by 26 strains and were presumptively considered to be *C. albicans* or *C. stellatoidia;* the remaining 12 were described as non-albicans *Candida*. All isolates were preserved and propagated by weekly subculture on Sabouraud's agar. All fungal strains in the culture collection showed catalase activity evidenced by the generation of bubbles when a colony from a Sabouraud's agar plate was placed in a drop of 3% hydrogen peroxide (certified 3%, Fisher, Fairlawn, NJ).

### Media Effect of Inhibition

The antifunal effect of hydrogen peroxide was tested in phosphate buffered saline (PBS) or in Sabouraud's broth (2% glucose, 1% primatone peptone, Sigma, St. Louis, MO). Dilutions of hydrogen peroxide (ranging from 0 to 17 mM/l) in both PBS and Sabouraud's broth were prepared. Two strains of *C. albicans* were grown overnight in Sabouraud's broth and each culture was diluted 1:10 prior to use in inoculation. One milliliter of each hydrogen peroxide dilution was inoculated with 10 μl of diluted starter culture containing about $10^4$ viable yeast cells verified by plate count. The dilutions were incubated at 37°C. After 2, 6, and 24 h of incubation, aliquots were removed from each tube and plated on Sabouraud's agar to determine the number of viable cells in each dilution. Because of the size of the experiment, only 2 yeast strains were evaluated.

### Minimal Inhibitory Concentration (MIC)/Minimal Cidal Concentration (MCC)

Hydrogen peroxide was added to Sabouraud's broth providing a range of concentrations from 4.4 to 176 mM/l and inoculated with approximately $10^4$ viable yeast cells, as described above. A control containing only Sabouraud's broth was also inoculated. After 24 h at 37°C, all tubes were evaluated by visual observation for turbidity, and the lowest concentration of hydrogen peroxide that prevented the development of turbidity was determined to be the MIC. A 10-μl aliquot removed from the tube showing the MIC endpoint was inoculated onto a Sabouraud's agar plate. If no growth was found on the plate, the MIC was considered to also be the MCC.

Germ-tube production provides a simple presumptive method of identifying *C. albicans,* although the less commonly isolated *C. stellatoidia* also germinates.[13] Germ-tube production was tested as described elsewhere[13] with the exception that it was done in a small volume. Each isolate was grown on a Sabouraud's agar plate. A single colony from each strain was combined with 100 μl of human serum in a microtiter well and incubated for 2–3 h. The content of each well was viewed microscopically and evaluated for the presence or absence of germ tubes.

The catalase activity was determined as described by Alcorn et al.[14] Briefly, a starter culture of yeast cells was grown on Sabouraud's agar and a heavy suspension made in PBS. An aliquot was removed for a plate count, and 100 μl of cell suspension was added to 1.8 ml of a 17-mM/l solution of hydrogen peroxide. The cells were allowed to stand for 15 min at room temperature and then centrifuged at 10,000*g* for 1 min to stop the reaction. The resulting supernatant was decanted into a quartz cuvette, and absorbance at 240 nm was determined and compared with the absorbance of a 17-mM/l solution of hydrogen peroxide. The catalase was expressed as units, in which 1 unit of catalase consumes 1 μM of hydrogen peroxide per minute at room temperature per $10^6$ viable yeast cells.

### RESULTS

To test whether *C. albicans* is intrinsically resistant to hydrogen peroxide, we randomly selected 2 isolates from the culture collection and incubated the organisms in dilutions of hydrogen peroxide in PBS ranging from 0 to 17 mM/l. Each dilution was inoculated with approximately $10^4$ viable yeast cells and incubated at 37°C. Plate counts were taken at 2, 6, and 24 h to determine the viability of the

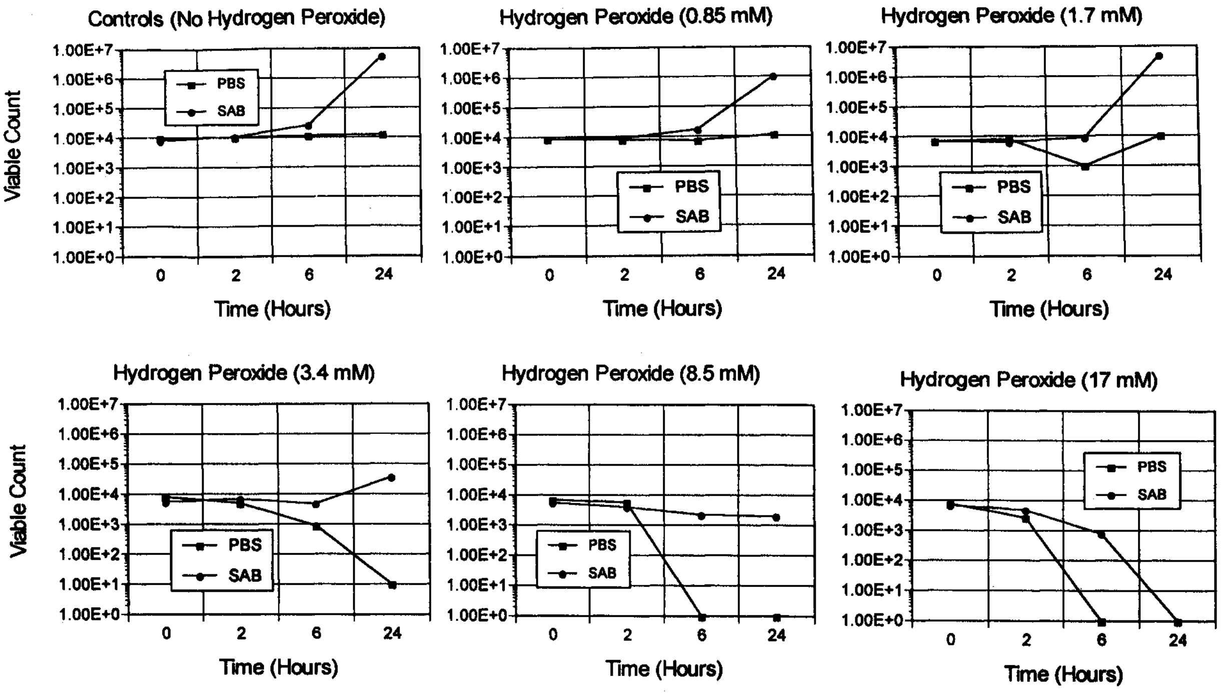

**Fig. 1. Time-dependent inhibition of a representative *C. albicans* strain in various concentrations of hydrogen peroxide. Hydrogen peroxide dilutions were prepared in growth medium (Sabouraud's broth) or PBS and inoculated with approximately 1 × $10^4$ viable yeast cells. The number of viable yeast cells at various sampling times is plotted on a logarithmic scale. Hydrogen peroxide in Sabouraud's broth was less effective in inhibiting yeast growth than equivalent concentrations in PBS. This experiment was repeated with a second yeast strain with similar results.**

cells. The results obtained for one of the isolates tested is shown in Figure 1. Because complex media components may alter the effectiveness of hydrogen peroxide on *C. albicans,* we simultaneously incubated the organisms in dilutions of hydrogen peroxide prepared in Sabouraud's broth and counted the viable yeast cells during the subsequent 24 h.The addition of undefined media to the incubation mixture mitigated but did not completely reverse the effect of the hydrogen peroxide (Fig. 1). We also noted that, despite the fact that hydrogen peroxide could be fungicidal, the effect was not instantaneous.

The results obtained from a second fungal strain (results not illustrated to avoid redundancy) showed the same trends of time-dependent susceptibility to hydrogen peroxide and diminished antifungal activity in the presence of growth media, although this second organism was slightly less susceptible to hydrogen peroxide, which suggested that individual strains may vary in their sensitivity to hydrogen peroxide (range of MIC values reported in Table 1).

TABLE 1. MIC data for all *Candida* isolates

| MIC (mM $H_2O_2$) | No. of strains inhibited | Mean catalase activity as units/million viable yeast cells (±S.D.) |
|---|---|---|
| 88 mM/l | 10 | 0.0104 (0.0146) |
| 17 mM/l | 26 | 0.0103 (0.0049) |
| 8.8 mM/l | 1 | — |
| 4.4 mM/l | 1 | — |

To determine which component of Sabouraud's broth was responsible for the diminished inhibitory effect of hydrogen peroxide, we tested the antifungal effect of hydrogen peroxide on 4 strains of yeast in the presence of PBS, PBS with 1% glucose or 2% peptone (primatone enzymatic meat hydrolysate, Sigma), or both. Peptone alone was much more effective than glucose in diminishing the inhibitory effect of hydrogen peroxide.

Having determined that the growth of *Candida* could be inhibited by sufficient concentrations of hydrogen peroxide, we next determined whether

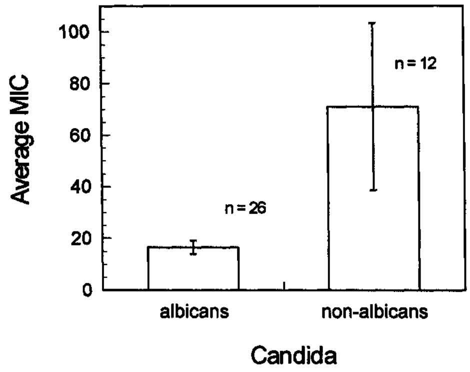

**Fig. 2. Susceptibility of 38 *Candida* strains to hydrogen peroxide. The average MIC (S.D.) for *C. albicans* expressed as mM/l is shown to be lower than the MIC for non-albicans *Candida*.**

the susceptibility to hydrogen peroxide was related to the catalase content of the organisms. As shown in Table 1, most isolates in our culture collection were susceptible to 17mM/l of hydrogen peroxide. For each yeast strain tested, an aliquot from the tube corresponding to the MIC was plated onto Sabouraud's agar to determine if the MIC was also the fungicidal endpoint. For most strains of *Candida,* a higher concentration of hydrogen peroxide was needed to produce a fungicidal effect than was required to prevent the development of turbidity. We also observed that the least susceptible organisms were the non-albicans *Candida*, as illustrated by Figure 2, which indicates that the average MIC for non-albicans *Candida* is nearly 4 times that for *C. albicans*. There was no apparent relationship between the amount of catalase measured and the susceptibility of individual strains (Table 1).

Although we demonstrated that media components could diminish the effectiveness of hydrogen peroxide on yeast, we anticipated that hemoglobin may have an even more profound effect on the antifungal capacity of hydrogen peroxide since it is considered to have a pseudocatalase activity. Although we anticipated that hemoglobin added to solutions of hydrogen peroxide may diminish its antifungal effect, we also wondered if growth of the organism in the presence of hemoglobin might alter the organism's susceptibility to hydrogen peroxide. An experiment was conducted with 8 representative yeast strains. Each test organism was grown overnight in either Sabouraud's broth or Sabouraud's broth with 1% bovine hemoglobin. MIC tests were prepared as described earlier and inoculated with the hemoglobin-adapted organisms or the non-adapted organisms. In addition, a set of hydrogen peroxide dilutions was prepared with 1% hemoglobin (final concentration) added and another set with 0.1% hemoglobin added. A 1% final concentration completely abrogated inhibition, even at 88 mM/l of hydrogen peroxide, but lesser concentrations of hemoglobin had a minimal effect, as shown in Table 2. When hemoglobin was added in a final concentration of 0.1%, the MIC of most strains was unaltered. Growth of the organisms in the presence of hemoglobin generally did not affect their susceptibility to hydrogen peroxide, as shown in Table 2.

All previous studies were performed in media with a neutral pH, whereas the pH of the vagina is more acidic. To predict how the vaginal pH might affect the hydrogen peroxide, we compared the antifungal activity of hydrogen peroxide against 4 strains of yeast at pH 4 and pH 7.2. We selected 2 yeast strains that had been shown to be quite sensitive to hydrogen peroxide and 2 that were less sensitive. For this study, we used PBS at pH 7.2 and PBS with the pH adjusted to 4.0. To these were added hydrogen peroxide in a final concentration of 44 mM/l for the least sensitive strains (strains W23 and W26) and 2.2 mM/l for the most sensitive strains (strains M47 and S6921). Each tube was inoculated with $1 \times 10^4$ viable yeast cells and incubated for 24 h at 37°C. Ten microliters was plated and the viable cells were counted (Figure 3), indicating that the antifungal effect of hydrogen peroxide at pH 4 was greater than at pH 7.2.

### DISCUSSION

The intricacies of the interactions among vaginal microorganisms and the underlying epithelium are being addressed by current research, which provides a partial explanation for the association of lactobacillus colonization and absence of vaginal symptoms. The production of bacteriocin, peroxide, and the elaboration of acidic metabolic endproducts may serve to limit to ability of other vaginal organisms to proliferate unimpeded. Nevertheless, many bacteria commonly found as members of the vaginal flora produce catalase which could eliminate hydrogen peroxide from the intravaginal milieu. The present investigation was un-

TABLE 2. Effect of bovine hemoglobin on the susceptibility of 8 *Candida* isolates to hydrogen peroxide

| Strain number of test organism | *Candida* grown in Sabouraud's broth without hemoglobin (mM/l) MIC of $H_2O_2$ without hemoglobin | MIC of $H_2O_2$ with 0.1% hemoglobin | *Candida* grown in Sabouraud's broth plus 1% hemoglobin (mM/l) MIC of $H_2O_2$ without additional hemoglobin |
|---|---|---|---|
| 47 | 88 | 88 | 88 |
| S6921 | 88 | 88 | 88 |
| 9324 | 320 | 88 | 88 |
| 8746 | 88 | 88 | 88 |
| 36 | 88 | 88 | 88 |
| W26 | 8.8 | 8.8 | 17.6 |
| 8749 | 8.8 | 8.8 | 8.8 |
| W23 | 35 | 35 | 35 |

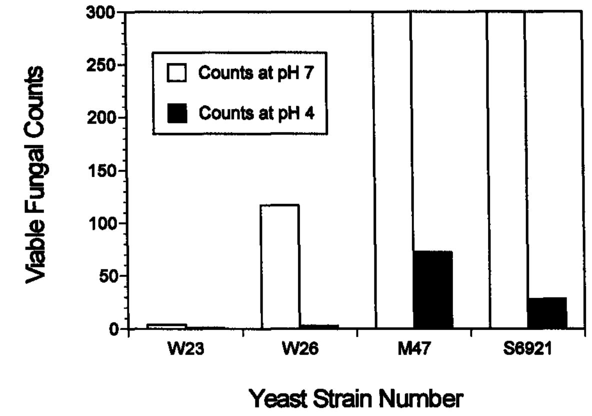

Fig. 3. Effect of pH on the susceptibility of 4 strains of *C. albicans* to hydrogen peroxide. Equal numbers of the test organisms listed were added to PBS, with the pH adjusted to pH 7.2 or pH 4, and hydrogen peroxide equal to one-half the MIC for each organism was added. After incubation for 24 h at 37°C, a 10-μl aliquot was removed and plated on Sabouraud's agar. The number of colonies from the 2 different pH values were plotted and, as shown by the figure, indicated that a lower pH accentuates the inhibitory effect of hydrogen peroxide.

dertaken to determine if a catalase-producing organism is intrinsically resistant to the antimicrobial effect of hydrogen peroxide.

We chose to study the effect of hydrogen peroxide on *Candida* because this organism is frequently found in vaginal cultures and is positive for catalase detected by the classical method of elaboration of bubbles by organisms added to a drop of 3% hydrogen peroxide. No correlation was found between the intrinsic catalase activity (measured as units per million viable yeast cells) and the concentration of hydrogen peroxide required to inhibit fungal growth in culture. Alcorn and coworkers[14] indicated that the susceptibility of *Neisseria gonorrhoeae* to hydrogen peroxide was not solely due to its catalase content, a finding similar to the present observations on *Candida* susceptibility.

From the small amount of hydrogen peroxide degraded by intact yeast cells, we inferred that intact yeast cells may have sufficient catalase to dispose of internally generated peroxide, but the enzyme is unavailable to destroy substantial quantities of extracellular hydrogen peroxide. The intense release of bubbles when a yeast colony is added to a drop of hydrogen peroxide reagent may be due to a disruption of the yeast cell integrity which releases significant catalase. This possibility was also suggested by a microscopic examination of the yeast cells exposed to 3% hydrogen peroxide (data not shown) which was consistent with yeast-cell damage.

The implication of these findings for the notion that peroxide-producing lactobacilli have a controlling influence on the vaginal flora is apparent. While it is impossible to extrapolate to the conditions in vivo, it appears that catalase production by *Candida* may not exempt them from growth restriction by hydrogen peroxide. In addition, hydrogen peroxide produced by vaginal flora organisms could exert antimicrobial effects through mechanisms other than the direct effect we examined in vitro. For example, Klebanoff and Smith[15] indicated that a hydrogen peroxide-peroxidase-halide system could be functional in the female genital tract. Other conditions extant in the vaginal microenvironment may also have an influence on the antimicrobial activity of hydrogen peroxide. For example, proteins present in the vaginal milieu may moderate the effect of hydrogen peroxide, and hemoglobin released during menstruation could significantly alter its influence. It has been previously observed that menstruation increases the prevalence of vaginal staphylococcal colonization[16] and the prevalence of various organisms is increased immediately postpartum.[17] Although these observations could be explained by the nutrient properties of blood, they could also be explained by the destruction of hydrogen peroxide by hemoglobin. Furthermore, while some conditions in the vagina could diminish the antimicrobial effect of hydrogen peroxide, others such as low pH could stabilize its effect.

The composition and control of the vaginal flora are not adequately explained by one simple aspect of the microenvironment, but the present study indicates that catalase production by vaginal bacteria may not preclude hydrogen peroxide from exerting a controlling influence on the normal flora.

### REFERENCES

1. Redondo-Lopez V, Cook RL, Sobel JD: Emerging role of lactobacilli in the control and maintenance of the vaginal bacterial flora. Rev Infect Dis 12:856–872, 1990.
2. Spiegel CA, Amsel R, Holmes KK (eds.): Diagnosis of bacterial vaginosis by direct Gram stain of vaginal fluid. J Clin Microbiol 18:170–177, 1983.
3. Weinstein L, Wawro NW, Worthington RV, Allen E: The influence of estrogenic hormone on the H-ion concentration and bacterial flora of the vagina of the immature monkey. Yale J Biol Med 11:141–148, 1938.
4. Weinstein L, Howard JH: The effect of estrogenic hormone on the H-ion concentration and the bacterial content of the human vagina, with special reference to the Doderlein bacillus. Am J Obstet Gynecol 37:698–703, 1939.
5. Klebanoff SJ, Hillier SL, Eschenbach DA, Waltersdorf AM: Control of the microbial of the vagina by $H_2O_2$ generating lactobacilli. J Infect Dis 164:94–100, 1991.
6. Eschenbach DA, Davick PR, Williams BL, Klebanoff SJ, Young-Smith K, Critchlow CM, Holmes KK: Prevalence of hydrogen peroxide-producing *Lactobacillus* species in normal women and women with bacterial vaginosis. J Clin Microbiol 27:251–256, 1989.
7. Nagy E, Petterson M, Mardh P-A: Antibiosis between bacteria isolated from the vagina of women with and without signs of bacterial vaginosis. AP Microbiol Immunol Scand 99:739–744, 1991.
8. Hillier SL, Krohn MA, Klebanoff SJ, Eschenbach DA: The relationship of hydrogen peroxide producing lactobacilli to bacterial vaginosis and genital microflora in pregnant women. Obstet Gynecol 79:369–373, 1992.
9. Ohm JM, Galask RP: Bacterial flora of the cervix from 100 prehysterectomy patients. Am J Obstet Gynecol 122: 683–687, 1975.
10. Larsen B: Normal genital microflora. In Keith LG, Berger GS, Edelman (eds): Common Infections. Lancaster: MTP Press, pp 3–32, 1985.
11. Tosado-Acevedo R, Toranzos GA, Alsina A: Extraction and purification of a catalase from *Candida albicans*. PR Health Sci J 11:77–80, 1992.
12. Nickerson WJ: Reduction of inorganic substances by yeast: Extracellular reduction of sulfate by species of *Candida*. J Infect Dis 93:43–56, 1953.
13. Lenette EH, Balows A, Hausler WJ Jr, Truant JP: Manual of Clinical Microbiology. 3rd Ed. American Society for Microbiology, Washington DC, pp 562–576, 1980.
14. Alcorn TM, Zheng H-Y, Gunther MR, Hassett DJ, Cohen MS: Variation in hydrogen peroxide sensitivity between strains of *Neisseria gonorrhoeae* is dependent on factors in addition to catalase activity. Infect Immun 62: 2138–2140, 1994.
15. Klebanoff SJ, Smith DC: Peroxidase-mediated antibacterial effect of rat-uterine fluid. Obstet Gynecol Invest 1:21, 1970.
16. Larsen B, Galask RP: Vaginal microbial flora: Composition and influences of host physiology. Ann Intern Med 96:926–930, 1982.
17. Goplerud CP, Ohm MJ, Galask RP: Aerobic and anaerobic flora of the cervix during pregnancy and the puerperium. Am J Obstet Gynecol 126:858–868, 1976.
